# Non-genomic transmission of longevity between generations: potential mechanisms and evidence across species

**DOI:** 10.1186/s13072-017-0145-1

**Published:** 2017-07-27

**Authors:** Alexander M. Vaiserman, Alexander K. Koliada, Randy L. Jirtle

**Affiliations:** 1 D.F. Chebotarev Institute of Gerontology, NAMS, Vyshgorodskaya st. 67, Kiev, 04114 Ukraine; 2 Department of Biological Sciences, Center for Human Health and the Environment, North Carolina State University, Raleigh, NC 27695 USA; 3 Department of Oncology, McArdle Laboratory for Cancer Research, University of Wisconsin-Madison, Madison, WI 53706 USA

**Keywords:** Epigenetics, DNA methylation, Transgenerational inheritance, Aging, Age-associated disease, Longevity

## Abstract

Accumulating animal and human data indicate that environmental exposures experienced during sensitive developmental periods may strongly influence risk of adult disease. Moreover, the effects triggered by developmental environmental cues can be transgenerationally transmitted, potentially affecting offspring health outcomes. Increasing evidence suggests a central role of epigenetic mechanisms (heritable alterations in gene expression occurring without changes in underlying DNA sequence) in mediating these effects. This review summarizes the findings from animal models, including worms, insects, and rodents, and also from human studies, indicating that lifespan and longevity-associated characteristics can be transmitted across generations via non-genetic factors.

## Background

Until recent years, a basic assumption in biology was that mutations in the DNA sequence were the only source of heritable phenotypic variation. Since the germ plasma theory of heredity was formulated by August Weismann more than a century ago [1], it is commonly believed that genetic information may be transmitted to the next generations by germ cells only, while somatic cells do not have any inheritance function. The core of this theory is the idea that information is not capable of being transferred from somatic to germline cells and, respectively, to the next generations. This concept is commonly referred to as the Weismann’s barrier. According to this concept, a strict distinction exists between innate and acquired characters. There is, however, significant empirical evidence to suggest that the Weismann’s barrier is not entirely impermeable and can be crossed [2, 3].

Examples for non-DNA sequence-based inheritance across generations have been obtained in a variety of species, including microbes, plants, worms, flies, fish, rodents, pigs, and humans [4, 5]. Despite a strong evidentiary base to demonstrate such a phenomenon, the possibility of the inheritance of acquired traits remains in the focus of intense debate today and raises numerous controversies in the scientific community. Accumulating evidence indicates that many effects (commonly referred to as ‘cross-generational’ or ‘transgenerational’ effects) can be transmitted across generations via non-genetic factors.

It should be taken into consideration that when discussing such effects in any species that the first generation’s primordial germ cells can be affected by in utero exposure. Thus, we must distinguish between parental effects triggered by certain environmental cues in the developing fetus, including the germline (i.e., intergenerational effects), from truly transgenerational effects induced in subsequent generations that were not exposed to the initial environmental triggers [6]. Most literature on transgenerational effects is focused mainly on maternal contribution to offspring phenotypes, while paternal effects are considered quite rare. In recent years, however, sufficient evidence has been reported that paternal contribution can be important as well [7].

**Fig. 1:**
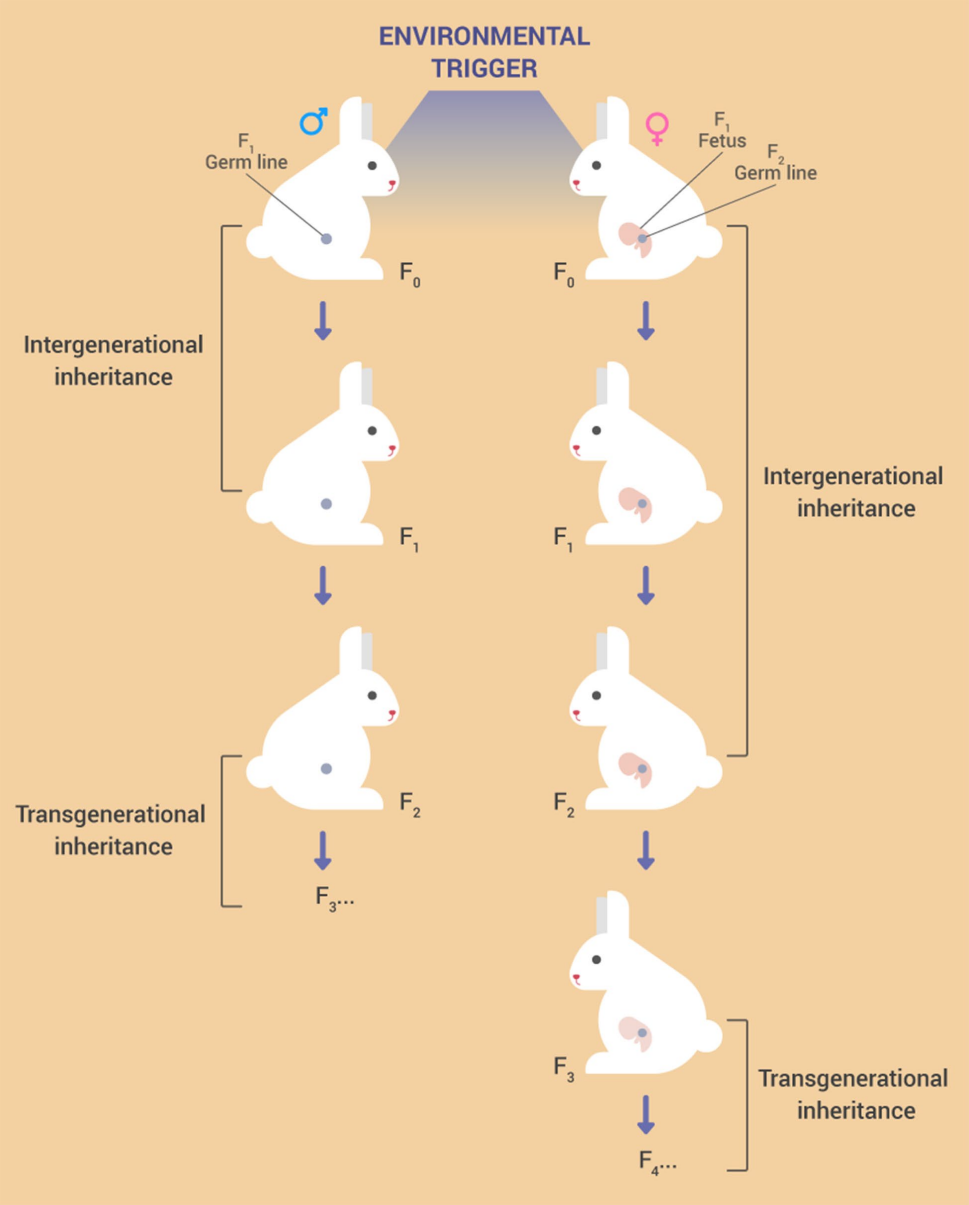
Distinction between intergenerational and transgenerational inheritance in mammals. In the case of intergenerational inheritance, parental exposures directly influence not only embryos and/or fetuses (F1 generation), but also already developing germ cells, giving rise to the F2 generation. Thus, F1 and F2 phenotypes may be directly exposed to external developmental cues, and F3 generation is the first one where the phenotype is not through primary triggering exposure. Therefore, true transgenerational effects include those that persist into the F3 generation [8]

A schematic representation of inter- and transgenerational effects is shown in Fig. 1.

Many recent papers highlight the role of epigenetic mechanisms in mediating these effects. These processes include modified patterns of DNA methylation and histone posttranslational modifications, replacement of canonical histones with histone variants, as well as altered noncoding RNA expression causing changed local accessibility to the genetic material and modified gene expression [9]. In a number of papers, the possibility of persistence of epigenetic modifications across multiple generations was reviewed and thoroughly discussed [10–14]. A good example of epigenetic intergenerational inheritance comes from the viable yellow agouti (*A* ^*vy*^) mouse model where maternal supplementation with methyl donors was shown to influence not only the affected dams, but also their F2-generation progeny via epigenetic modifications in the germline [15, 16].

In several recent studies, the potential importance of non-genomic transgenerational effects in the inheritance of age-related characteristics has been highlighted [6]. In particular, the evidence for transgenerational effects of maternal diet on reproductive and metabolic aging in offspring has been obtained in various mammalian species such as guinea pigs, rodents, and sheep [17]; however, the transgenerational effects on longevity have been reported only rarely to date. Most of the papers reviewing and discussing such effects are focused solely on data obtained from the nematode *Caenorabditis elegans* [18–21], although similar findings were obtained in other species as well (see subsections below). In this review, evidence from various taxa demonstrating that lifespan and longevity-associated characteristics can be transmitted across generations is described and discussed.

## Basic mechanisms of epigenetic regulation

Epigenetic modifications refer to both mitotically and meiotically heritable alterations in gene expression that occur without changes in underlying DNA sequence [22]. While DNA is relatively static throughout ontogenesis, the epigenetic code changes dramatically during the embryonic development of the organism to initiate differential gene expression patterns between various developing tissues. This code consists of chemical modifications of DNA and histone proteins that play a crucial role in packing the DNA by forming nucleosomes.

In many species including mammals, DNA methylation, consisting of the addition of a methyl group at the fifth position of cytosine followed by a guanine nucleotide (a CpG dinucleotide), is one of the most important mechanisms of epigenetic regulation [23, 24]. This process mostly occurs within so-called CpG islands, i.e., regions of the genome that are rich in cytosine followed by guanine nucleotides. CpG islands are typically common near transcription start sites often associated with promoter regions. Methylation of CpG islands in promoter regions of various genes usually leads to their transcriptional silencing, although several transcription factors important for cell reprogramming during development were recently identified that prefer to bind to CpG-methylated sequences [25].

Posttranslational modification of core histone proteins, including the acetylation, phosphorylation, methylation, phosphorylation, sumoylation, and ubiquitination of histone tails, is another key mechanism of epigenetic regulation [26]. Various combinations of histone modifications form the histone code for an organism which mark the functional units of chromatin, recruiting transcription factors, and coactivators/cosuppressors that regulate chromatin structure and gene activity. Among all histone modifications, methylation and acetylation of histone tails appear to be the most important modifications linked to gene expression. Transcriptional activity can be affected by histone modifications through two major mechanisms. Firstly, they can change the structure and conformation of chromatin. Secondly, they can provide signals for particular enzymes to recruit transcriptional activators or suppressors. The dynamic “writing” and “erasing” of histone modifications are conducted by specific enzymes that catalyze the processes of the addition or removal of acetyl groups from lysine residues on the histone N-terminal tails. The main “writers” include histone acetyltransferases and methyltransferases, while “erasers” include histone deacetylases and lysine demethylases [26, 27].

More specifically, histone acetyltransferases acetylate lysine residuals by transferring acetyl groups from acetyl coenzyme A (acetyl-CoA) to form ε-*N*-acetyl lysine. This modification leads to the neutralization of the positive charge of lysine and can consequently disrupt the interactions between the DNA and the histone tails. Acetylated histones are commonly associated with euchromatin and active transcription. In contrast, deacetylation of histones results in restriction of DNA accessibility through revealing the positive charge of lysine. This permits interactions between the DNA and the histone tails and results in chromatin compaction. Similarly, the phosphorylation of threonine, serine, and tyrosine by kinases and phosphatases alters histone’s net charge, thereby contributing to the modification of the chromatin structure. The processes of DNA methylation and histone modification are closely interconnected with each other. DNA methylation can affect the histone modification and vice versa, thereby collectively influencing chromatin accessibility to RNA polymerase and transcription factors.

Recently, one more key component of epigenetic control of gene expression by noncoding RNAs has been discovered. Noncoding RNAs can regulate gene activity at both the transcriptional and posttranscriptional levels [28, 29]. They are functional RNA molecules that are transcribed from DNA but not translated into proteins. Several noncoding RNAs may interfere with the functionality of messenger RNAs (mRNAs) through a mechanism of RNA interference. By this mechanism, gene expression is regulated in a sequence-specific way without changing target sequences. The microRNAs (miRNAs), small (18–22 nucleotides in length) RNA molecules that may negatively regulate posttranscriptionally the expression of their target genes, are the most thoroughly studied class of noncoding RNAs involved in epigenetic regulation. The miRNA “seed sequences” (nucleotides 2–8 at the 5′ end) bind to complementary sites in the 3′ untranslated region of the target mRNA and inhibit the translation of this mRNA or cause its degradation. This results in the premature degradation or in the stop of translation due to the formation of so-called RNA-induced silencing complexes (RISCs). The miRNAs commonly have multiple targets, and a particular gene may be targeted by different miRNAs. Thus, signaling pathways regulated by these molecules may be exceptionally complex. Importantly, the expression of miRNAs can be modulated by DNA methylation or histone modifications and vice versa, thereby leading to regulatory feedback loops in epigenetic regulation. Recently, the crucial role of noncoding RNAs in transgenerational epigenetic inheritance has been highlighted [3].

An important point in terms of transgenerational epigenetics is that, in mammals, epigenetic marks such as DNA methylation are nearly globally erased and then re-established through two waves of demethylation followed by *de novo* methylation during the stages of preimplantation and primordial germ cell (PGC) maturation [30, 31]. It is traditionally believed that such re-establishment of epigenetic signatures encompasses the entire genome, except for imprinted genes [30], and prevents the transmission of acquired epigenetic information across generations. Nevertheless, some methylation marks are, in fact, not fully erased, and persist throughout these periods. For example, in the Guo et al. study [32], 7.8 and 6.0% of the methylation marks in male and female PGCs, respectively, were shown to be still not erased 10–11 weeks after gestation (i.e., a time of minimal DNA methylation).

It has been repeatedly shown that the epigenome (i.e., the totality of epigenetic marks across the entire genome) demonstrates its highest sensitivity to environmental cues during specific windows of sensitivity in early development when the process of re-establishment of epigenetic marks takes place [33], and that epigenetic transgenerational inheritance operates during these same periods [4]. Moreover, the obvious distinction between epigenetic (‘soft’) and genetic (‘hard’) inheritance is that epigenetic changes, unlike the genetic ones, are reversible and can persist for only a few generations in the absence of the inducing trigger [10]. Therefore, it is commonly believed that attenuation of epigenetic memory over a multiple generations is a passive process attributable to dilution of particular inherited factors [34].

**Fig. 2:**
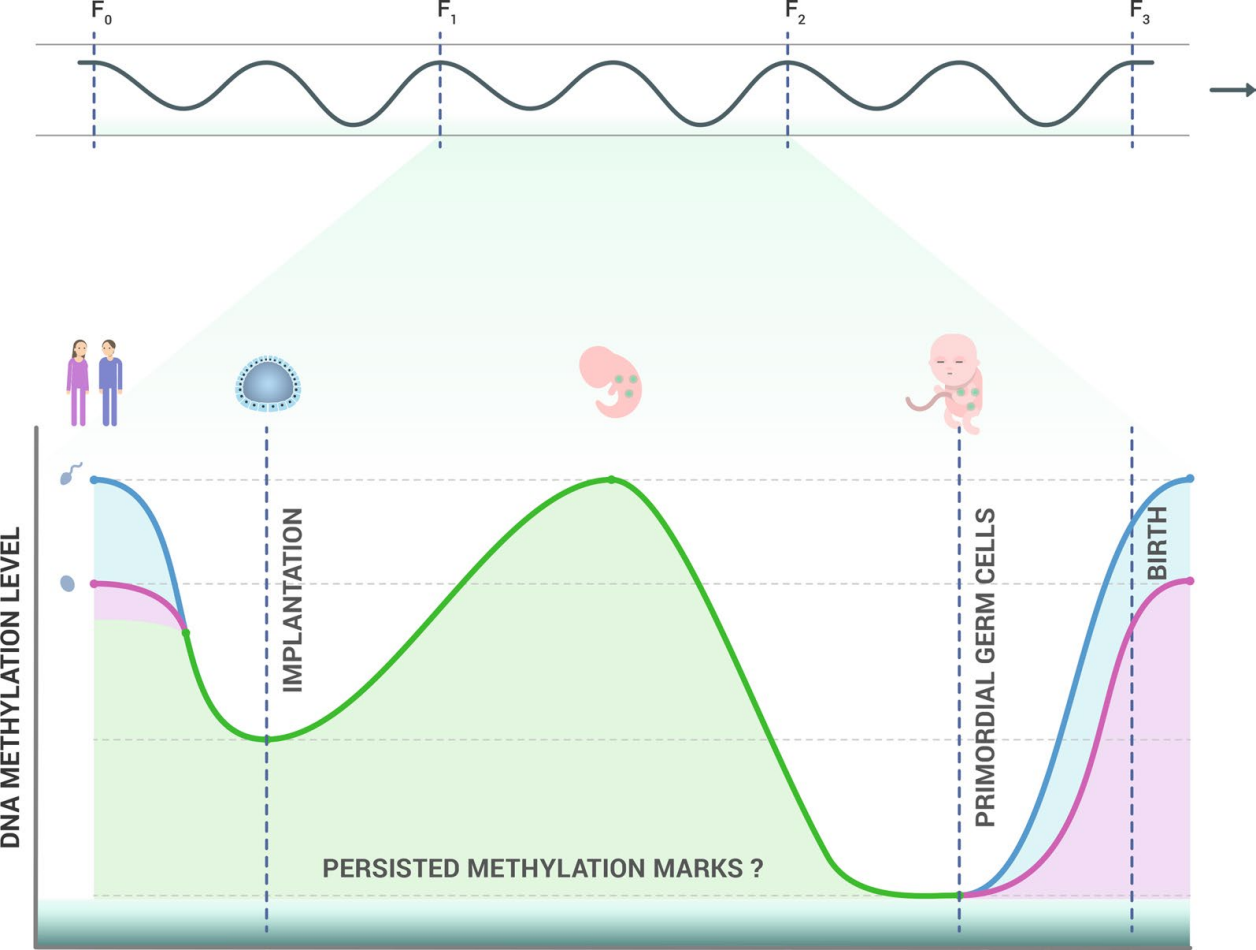
Genome-wide demethylation and *de novo* DNA methylation processes in the mammalian germline and in preimplantation embryos. The levels of global DNA methylation are indicated on the y-axis and are shown by a blue line for the paternal genome and a pink line for the maternal genome. The top bar schematically presents waves of demethylation followed by *de novo* methylation in F0–F3 generations

**Fig. 3:**
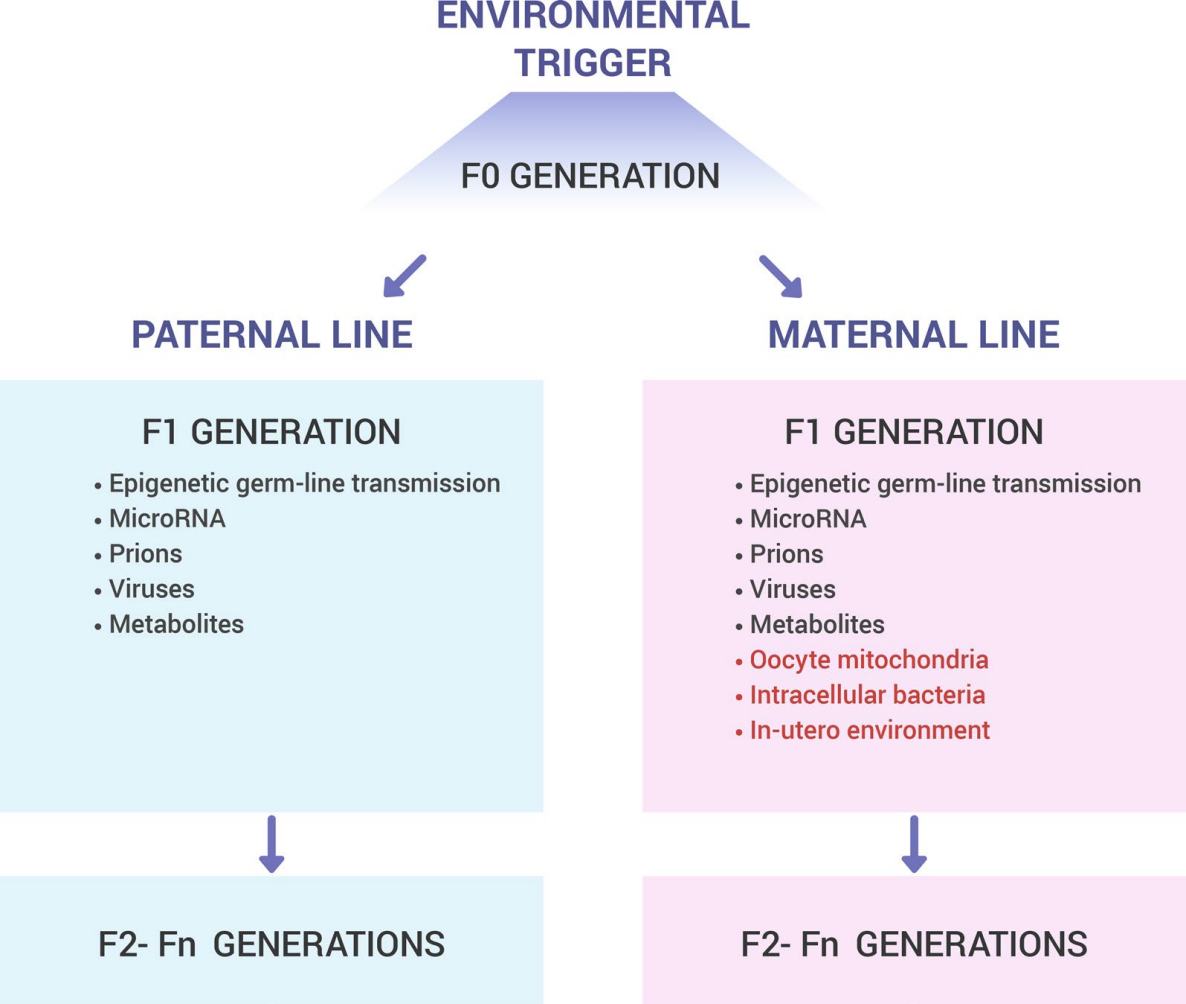
Potential mechanisms of transgenerational transmission of environmentally induced effects through both maternal and paternal lines (*black letters*) or only the maternal line (*red letters*). The figure is based on hypothetical mechanisms suggested by Heard and Martienssen [90] to explain transgenerational inheritance of acquired information

It can be speculated that preservation of these marks might be limited not only to imprinted regulatory elements referred to as imprintome [35], but also to several non-imprinted genes [36]. Moreover, the imprinted loci are environmentally sensitive as well, especially during early developmental stages. Thus, imprinted gene expression is not only parent-of-origin dependent, but is also influenced by environmental exposures early in life [37]. The retention of methylation marks in both imprinted and non-imprinted genes would allow for the transmission of epigenetically acquired memory to the later germ cells, thereby being one of the mechanisms potentially involved in the process of transgenerational epigenetic inheritance. A schematic representation of the dynamics of genome-wide DNA methylation reprogramming during the early mammalian development is presented in Fig. 2. Other mechanisms potentially contributing to this process are presented in Fig. 3.

## Findings from animal studies

Several recent findings indicate a resistance of epigenetic marks to complete erasure during these developmental periods, allowing their transmission across generations [13, 38]. Examples of inter- or transgenerational transmission of epigenetic marks have been reported for many taxa from microorganisms to humans [5]. In the subsequent subsections, evidence for inter- and transgenerational effects on lifespan and longevity-associated traits across species is summarized and discussed.

### Nematode worm

Transgenerational epigenetic inheritance of longevity in *Caenorhabditis elegans* was revealed in elegant research by Greer et al. [39]. In this study, the lifespan in four consecutive generations of nematodes was found to be strongly affected by a specific histone modification, namely by a change in the histone H3 lysine 4 trimethylation (H3K4me3) complex in the ancestral generation. The authors used crosses between long-lived mutant females having the modified H3K4me3 complex with wild-type males to obtain the heterozygous offspring F1 generation. Descendent F2 generations (both homozygous mutant or wild type) were generated by self-fertilization of heterozygous F1 hybrids. F3- to F5-generation offspring were produced in a similar manner. Surprisingly, the genetically wild-type F2–F4 progeny expressed long-lived phenotype similar to mutant controls. This effect was observed up to the F5 generation, when the normal lifespan was restored. The genome-wide transcriptional analysis demonstrated that both long-lived mutant and wild-type F2-F4 offspring shared a substantial subset of differentially regulated genes with long-lived ancestors.

More recently, transgenerational effects on postmitotic aging of somatic cells as well as proliferative aging of germ cells were also observed in *C. elegans* mutant for H3K4 demethylases, RBR-2, and SPR-5 [40]. Deletion of the H3K4me2 demethylase, SPR-5, resulted in a transgenerational increase in *C. elegans* longevity [41]. Such transgenerational extension of lifespan has been found to be dependent on a hormonal signaling pathway comprising the steroid dafachronic acid, which is an activator of the nuclear receptor *DAF*-*12*. On the basis of these data, the authors concluded that loss of the demethylase SPR-5 leads to misregulation of H3K4me2 and activation of a particular longevity-regulating signaling pathway, thereby resulting in a transgenerational lifespan extension. Intergenerationally inheritable survival advantages induced by low-level stress exposures during developmental stages in *C. elegans* were also found in the study by Kishimoto et al. [42]. In this research, factors such as the insulin/insulin-like growth factor signaling effector DAF-16/FOXO and the heat-shock factor (HSF)-1 were substantially involved in the formation of epigenetic memory in the parental somatic cells, and this memory was maintained by H3K4me3 in the germline across generations.

In discussing the mechanisms underlying the transgenerational effects on longevity in *C. elegans*, Pang and Curran speculated that such effects can be explained by transgenerational epigenetic inheritance via signaling regulators, transcription factors, or miRNAs, which might influence the expression of particular longevity-associated genes [19]. Lim and Brunet also suggested the potential importance of miRNAs or specific transcription factors in observed transgenerational effects, although the impacts of other factors, such as prions, cannot be excluded [10].

### Fruit fly

Inter- and transgenerational effects on lifespan were also observed in the fruit fly, *Drosophila melanogaster*. In research by Buescher et al., a high-sugar maternal diet altered body composition of offspring at the larval stage for at least two generations, causing development of an obese-like phenotype in adult offspring maintained under suboptimal (high-calorie) nutritional conditions [43]. The expression levels of metabolic genes known to be strongly associated with longevity were substantially modified in the offspring of affected flies. In a study by Xia and de Belle [44], transgenerational effects on longevity and reproduction were obtained by post-eclosion nutritional manipulations with low-, intermediate-, or high-protein contents. Both low-protein and high-protein diets shortened lifespan, while the intermediate-protein diet considerably extended lifespan up to the F3 generation. Intergenerational effects were also revealed with respect to female reproductive activity (fecundity). It was reduced in the low-protein groups and enhanced in the intermediate-protein groups throughout F0–F2 generations. In subsequent research by the same authors, exposure to a low-protein diet during the post-eclosion adult life led to the upregulation of the H3K27-specific methyltransferase, E(z), thereby causing enhanced levels of H3K27 trimethylation (H3K27me3) [45]. These changes in H3K27me3 levels were accompanied by a shortening of longevity in F0 generation as well as in the F2 offspring. Both specific RNAi-mediated knockdown of E(z) or pharmacological inhibition of its enzymatic function by a histone methyltransferase inhibitor Tazemetostat (EPZ-6438) resulted in lower levels of H3K27me3 across generations. Furthermore, Tazemetostat completely mitigated the lifespan-reducing effect of the parental exposure to a low-protein diet. In a recent study by Roussou et al. [46], dietary restriction in adult *Drosophila* males resulted in life extension in their F2 offspring.

Intergenerational effects on longevity were also observed in *Drosophila* exposed to factors other than alterations in nutrition, although epigenetic changes accompanying these processes were not identified in these studies. Both cross-life stage and cross-generational adaptive plasticity induced by gamma irradiation at the egg stage were found by Vaiserman et al. [47]. Such exposure resulted in enhanced resistance to the heat shock and starvation stresses and also in extended lifespan in the F0 and F1 generations. Intergenerational impact of gamma irradiation in the ancestral F0 generation on the flies’ longevity in the F1 and F2 generations was also demonstrated by Shameer et al. [48]. Adult paternal exposure to small-to-moderate doses of radiation (1–10 Gy) resulted in increased lifespan of both male and female offspring, while exposure to high doses (40 and 50 Gy) decreased the descendants’ longevity. These effects disappeared in the F3 generation.

### Rodents

There is consistent evidence from both mice and rat models that modulating the maternal dietary status such as protein restriction during pregnancy can significantly affect lifespan [49] as well as profiles of expression of many longevity-associated genes [50–52] in F1 offspring. Nevertheless, there are only a few studies that directly investigate inter- and transgenerational effects on longevity in rodents, for the obvious reason that they live much longer than short-lived models such as nematodes or flies. Therefore, most evidence supporting the possibility of inter- and transgenerational effects on longevity in rodents is indirect.

Evidence of cross-generational epigenetic inheritance of longevity-associated characteristics has been observed in several mouse models mainly focused on epigenetic processes contributing to metabolic regulation, which is known to play a critical role in determining longevity potential [53]. For instance, in utero exposure to maternal obesity contributed to lifespan-limiting health problems in future generations, including increased rates of metabolic syndrome, type 2 diabetes, and cardiovascular disease [54]. In the mouse model, intergenerational environmental reprogramming of the expression of key metabolic genes in response to the paternal diet was also observed [55]. Descendants of males fed with low-protein diet demonstrated enhanced hepatic expression of a number of genes that contribute to cholesterol and lipid biosynthesis, as well as decreased levels of cholesterol esters, in comparison with the offspring of males fed a control diet. Furthermore, moderate (~20%) DNA methylation changes were induced by the paternal low-protein diet, including changes in methylation of the key regulator of lipid metabolism, *Ppara*. In another mouse study, the induction of a prediabetic state in male ancestors by combination of streptozotocin and high-fat diet caused glucose intolerance, insulin resistance and enhanced diabetes risk in their F1 and F2 offspring [56]. These effects were accompanied by modulations in the expression levels of genes related to glucose metabolism and insulin signaling in pancreatic beta cells as well as by altered methylation of these genes. Moreover, the offspring of prediabetic parents demonstrated substantially modified characteristics in the sperm methylome. These alterations markedly coincided with those found in beta cells.

Nutritional stresses experienced by male ancestors throughout critical stages of development (i.e., prior to breeding) also affect the metabolic health of their offspring. For example, preconceptional paternal food deprivation resulted in a consistent decrease in average serum glucose levels as well as significant changes in insulin-like growth factor-1 (IGF-1) expression in both male and female offspring [57]. F1 male mice exposed to maternal undernutrition during prenatal life produced F2 male offspring with impaired glucose tolerance and increased adiposity [58]. These intergenerational metabolic changes were shown to be mediated by alterations in the germline DNA methylome [59].

In the Ding et al. study, an impaired glucose tolerance was observed in both F1- and F2-generation offspring of animals with gestational diabetes mellitus [60]. These intergenerational adverse health outcomes were accompanied by down-regulated expression of the imprinted genes, *Igf2* and *H19*, caused by abnormal methylation patterns in the differentially methylated imprint control regions of these genes in pancreatic islets. In addition, modified expression of *Igf2* and *H19* genes was demonstrated in the sperm of adult F1 offspring of female mice with gestational diabetes. These findings provide evidence that epigenetic alterations can be imprinted in germ cells and contribute to this type of intergenerational transmission.

Transgenerational inheritance of characteristics associated with longevity and susceptibility to lifespan-limiting pathological conditions was also observed in several rat studies [61]. Most of these studies used maternal protein restriction during pregnancy as an affecting factor. Such prenatal exposure in the F0 generation resulted in inter- and transgenerational effects in F1–F3 generations. Both elevated blood pressure and impaired endothelial function were found to be passed through the maternal line to the F2 generation in the absence of any additional nutritional challenges to the F1 mothers [62]. Exposure to maternal low-protein diet during gestation led to impaired nephrogenesis and hypertension in the F2 generation. These effects were shown to occur in both the maternal and paternal lines [63]. Protein restriction during gestation and/or lactation also resulted in unfavorable intergenerational effects on biometric parameters (i.e., body mass and fat mass) and on glucose, insulin, and leptin metabolism, thereby causing insulin resistance in F1 and F2 adult rat progeny [64]. Global maternal dietary restriction during pregnancy resulted in hypertension and endothelial dysfunction in F1–F3-generation offspring [65]. Intergenerational cardio-renal effects were also observed in a rat model of utero-placental insufficiency. When compared to sham surgery, bilateral uterine vessel ligation in the ancestral generation led to elevated systolic blood pressure in F2 males 6–9 months of age, but not female offspring [66].

Higher risk of disorders such as kidney and immune pathologies, and infertility and cancer were also found in rats exposed to non-dietary factors (i.e., toxicants). For example, exposure to vinclozolin, a fungicide with anti-androgenic activity, throughout critical periods of embryonic development significantly influenced disease susceptibility in the affected generation as well as their offspring in over four successive generations [67]. Such transgenerational effects were accompanied by modified characteristics of DNA methylation of certain genes in the sperm that persisted across several generations. The exposure of pregnant rats to the insecticide, dichlorodiphenyltrichloroethane (DDT), caused the formation of prostate, kidney, and ovary disorders, as well as tumor development in adult animals of the F1 generation [68]. In the F3 generation, obesity and obesity-related pathologies occurred in more than 50% of the male and female offspring. Most of the genes associated with DDT-induced differentially methylated DNA regions were previously found to be associated with obesity.

Thus far, only one study provides direct evidence that rodent longevity can be transmitted across generations. In this study, dietary restriction during preconceptional or both preconceptional and gestational periods resulted in increased body weight and life shortening in the second-generation male, but not the female offspring [69].

## Findings from human studies

Evidence for the existence of intergenerational and transgenerational inheritance of longevity and lifespan-limiting conditions in human populations is obtained from epidemiological and demographic studies. For instance, the F2-generation offspring of women who were exposed to the famine of 1944–1945 in the Netherlands (i.e., Dutch Hunger Winter) throughout the gestational period had 1.8 times more health problems in their adulthood than descendants of non-exposed women [70]. The offspring of the fathers, but not the mothers who suffered from intrauterine undernutrition during the Dutch famine also had a significantly greater weight and body mass index in their adult life than the descendants of parents unexposed to the famine [71]. The offspring of parents born during the famine in China from 1959 to 1961 (i.e., Great Leap Forward Famine) were significantly shorter (i.e., boys by 1.9 cm and girls by 1.8 cm) than children whose parents were not exposed to starvation during the famine period [72]. The authors speculated that such anthropometric changes could be associated with negative impacts on the life-course health status of the offspring of famine-exposed parents. Similar effects were also revealed in a Tunisian study where lower height, weight, and smaller placental size (i.e., conditions known to be associated with elevated risk of adult cardio-metabolic pathologies) were observed in the infant offspring of mothers born during Ramadan fasting [73]. The potential role of epigenetic modifications in both parental and grandparental generations in the risk of familial cancers has been also demonstrated [74].

More direct evidence for the possibility of intergenerational inheritance of human longevity was provided in research carried out in the Överkalix, an isolated community in Northern Sweden. In human cohorts born in this region in 1890, 1905, and 1920, the transgenerational consequences were studied of the ancestor’s nutrition during their slow growth period (i.e., 9–12 years) when there is increased susceptibility to environmental influences [75–78]. In these studies, it was shown that nutrition of the paternal ancestors during the slow growth period significantly influenced the mortality rate and life expectancy of their grandchildren. If the availability of the food was limited during the father’s slow growth period, the descendant’s cardiovascular mortality was low, while overeating of the paternal grandfathers during this period resulted in a fourfold increase in diabetes mortality in their grandchildren [76]. Moreover, these intergenerational effects were gender specific. The paternal grandmother’s nutrient supply affected granddaughter’s mortality risk, whereas the paternal grandfather’s nutrient supply was associated with the mortality risk in the grandsons [77–79]. More recent research by the same group showed that abrupt changes in dietary characteristics among successive years throughout the period of slow growth in both maternal grandparents, as well as in paternal grandfathers, do not affect cardiovascular mortality of their offspring. If, however, the paternal grandmothers were exposed to sharp nutritional changes during the period preceding puberty, their son’s daughters had a 2.7-fold higher risk for cardiovascular mortality than descendants of unexposed grandmothers [80]. Selection, as well as learning or imitation, was thought to be unlikely explanations for the observed associations. The authors concluded that X-linked epigenetic transmission through spermatozoa provides a plausible explanation for this effect.

In another Swedish study, where illegitimacy was used as an indicator of socioeconomic adversity in early life, the adult mortality rates in both males and females born outside the marriage at the beginning of the twentieth century were significantly higher than those of persons born in wedlock [81]. The male offspring of illegitimate parents were also less likely to live until age 80 than male descendants whose biological parents were married. Similar trends were observed in their children and grandchildren as well. Results indicating that social disadvantages in ancestral generations contribute to health disadvantages during the subsequent two generations were also observed in another study by the same group [82]. In sons and grandsons of men born outside wedlock, a 1.64- and 1.83-fold excess risk of circulatory disease, respectively, was observed. The impacts of the maternal and paternal grandfather were approximately equal in magnitude.

Intergenerational effects triggered by factors other than nutrition have also been demonstrated. For instance, paternal smoking during the slow growth period (i.e., <11 years of age) was significantly associated with greater body mass index at the age of 9 in sons, but not in daughters [78]. Northstone et al. also showed that paternal smoking before puberty is associated with an increased risk of obesity in the adolescent offspring [83].

## Conclusion and perspectives

Non-genomic transgenerational inheritance has been described repeatedly across species and appears to be a general biological phenomenon. In evolutionary terms, the transmission of the adaptive transcriptional patterns acquired throughout the parental life course in subsequent generations via the mechanism of epigenetic memory can enable the organism to better survive in potentially adverse environments [84]. In particular, it has been repeatedly reported that offspring of parents exposed to nutritional stresses exhibit altered expression of genes related to metabolic functions including those implicated in pro-longevity metabolic pathways [6]. The mechanisms potentially responsible for such inter- and transgenerational effects are currently the subject of active investigation [5, 14, 85–89]. In most studies on short-lived models such as nematodes and flies, the role of histone modifications in transgenerational transmission of epigenetic information was highlighted, while in rodent models changes in DNA methylation have been mainly detected.

An important outstanding question is how the phenotypic information originating in somatic cells can be transmitted to germ cells to induce epigenetic changes in the germline. Recently, exosome-like miRNA-bearing extracellular vesicles shed by somatic cells have been proposed as potential candidates for the transmission of environmentally induced epigenetic changes in the germline [2]. The possibility of transgenerational transfer of acquired information is definitely much more complex if implemented via the maternal line because it could include not only epigenetic modifications in gametes but also programming events through the egg cytoplasm, the environment of the reproductive tract, and also changes in maternal physiology adapting inadequately to pregnancy demands [17]. Importantly, however, not only maternal but also paternal environmental exposures have been shown to induce epigenetic changes that pass through the male germline to affect health status in the offspring [7, 91].

A schematic representation of basic molecular mechanisms potentially responsible for inter- and transgenerational effects contributing to longevity is presented in Fig. 3.

The persistence of epigenetic memory about environmental stresses between generations is likely a more common phenomenon among short-lived species such as nematode worms than among long-lived species. Nevertheless, there exists evidence that epigenetic transgenerational effects can influence life expectancy and longevity-associated traits also in mammals, including humans as well. Epigenetic memory about lifestyles such as unhealthy diet, smoking, or substance abuse in the ancestral generation can likely affect the health status across several successive generations. These processes can also influence disease susceptibility and aging phenotypes, thereby affecting longevity across generations. Although most examples of cross-generational non-genomic inheritance are for adverse effects or pathological conditions, there is also evidence that exposure to mild stressors in early development can result in beneficial (hormetic) effects and that adaptive modulation of epigenetic processes could significantly contribute to these effects [92–94]. There is also evidence that adaptive/hormetic effects can persist over several generations [42, 47, 48].

An investigation of inter- and transgenerational effects in human populations presents a number of research challenges [95]. The important issue is uncertainty in accurate determination of the timing and level of exposure, as well as very long generation times in human cohorts [96]. Quasi-experimental designs could likely be useful to overcome these problems. Such research designs or “natural experiments” allow for the identification of cohorts where exposure is relatively homogeneous across the whole population and timing of exposure can be accurately determined by historical records. Good examples are studies of long-term health consequences of severe famines in Holland in 1944–1945 (*‘Dutch Hunger Winter’*) [97] and in Ukraine in 1932–1933 (*‘Holodomor’*) [98]. It is likely that further insights into the possibility of the transmission of non-genomic information across human generations will be gained through such research designs when studying the descendants of persons exposed to hunger during these historical events. In the future, transgenerational effects could become much more suitable to investigation in human populations owing to the utilization of well-characterized prospective birth cohorts which have been established in many countries worldwide. An important point is that it is possible to obtain biological samples for epigenetic analyses from such studies [95].

In order to estimate the potential impact of epigenetic memory in determining life expectancy, further research should answer several key outstanding questions presented in Box.

**Table:** 

Box: Outstanding questions
• What is the relative contribution of the epigenetic inheritance in comparison with the genetic one?
• Is transgenerational epigenetic inheritance widespread across species?
• How is phenotypic information originating in somatic tissues transmitted to germ cells?
• How many generations can epigenetic memory persist?
• Why do non-genomic effects disappear over a few generations?
• Could transgenerational epigenetic processes influence genetic inheritance, and vice versa?
• Are mechanisms responsible for transgenerational non-genomic effects the same across species?

Answering these questions could provide new insights into the origin of the phenomenon of transgenerational epigenetic inheritance. Potential reversibility of unfavorable epigenetic signatures can provide a promising approach to designing novel therapeutic interventions [99, 100]. Overcoming the inertia caused by the persistence of epigenetic effects induced in previous generations may become in the future an important component of effective prevention of human diseases [101, 102]. Various nutritional and pharmacological epigenetic modulators have already demonstrated their therapeutic potential for diminishing or reversing unfavorable epigenetic alterations [103, 104]. This can be important not only to correct epigenetic changes directly induced by unfavorable environmental exposures, but also to eliminate epimutations inherited from previous generations. Thus, broader implementation of epigenome-targeted therapeutic interventions may hold great promise to improve the health and longevity in human populations across the globe.

## Abbreviations

CpG: a cytosine followed by a guanine nucleotide
DDT: dichloro-diphenyl-trichloroethane
H3K9me3: histone H3 lysine 9 trimethylation
H3K27me3: histone H3 lysine 27 trimethylation
miRNA: microRNA
Mtrr: methionine synthase reductase
PGC: primordial germ cell
RISC: RNA-induced silencing complex
